# Primary care and healthcare utilization among older Brazilians (ELSI-Brazil)

**DOI:** 10.11606/S1518-8787.2018052000595

**Published:** 2018-10-25

**Authors:** James Macinko, Fabíola Bof de Andrade, Paulo Roberto Borges de Souza, Maria Fernanda Lima-Costa

**Affiliations:** I UCLA. Fielding School of Public Health. Departments of Health Policy and Management and Community Health Sciences. Los Angeles, CA, USA; II Fundação Oswaldo Cruz. Instituto René Rachou. Programa de Pós-Graduação em Saúde Coletiva. Belo Horizonte, MG, Brasil; III Fundação Oswaldo Cruz. Instituto René Rachou. Núcleo de Estudos em Saúde Pública e Envelhecimento. Belo Horizonte, MG, Brasil; IV Fundação Oswaldo Cruz. Instituto de Comunicação e Informação Científica e Tecnológica em Saúde. Rio de Janeiro, Brasil

**Keywords:** Aged, Health Services for the Aged, Health Services Needs and Demand, Primary Health Care

## Abstract

**OBJECTIVE:**

To characterize healthcare access and utilization among older Brazilians.

**METHODS:**

Data are from the baseline wave of the Brazilian Longitudinal Study of Aging (ELSI-Brazil), which is a nationally representative, population-based cohort study of persons aged 50 years and older conducted in 2015/2016 (n = 9,412). The prevalence of barriers to primary care and number and type of doctor visits in the past 12 months are compared by three main sources of healthcare (private, Family Health Strategy, traditional public clinics). Two-part multivariable hurdle analyses assess the relation between healthcare utilization, primary care problems, and source of healthcare, while controlling for healthcare determinants.

**RESULTS:**

Females comprised 54% of the sample, with a mean age of 63 years. There were no demographic differences by source of healthcare. Nearly 83% had at least one doctor visit in the past 12 months, with higher use among private health plan holders. Private health plan holders most frequently visited specialists, while those using the public system were more likely to visit a general practitioner. Primary care barriers averaged 3.5 out of 12 and were the highest among those using traditional health posts. A greater number of primary care problems was negatively associated with all types of healthcare utilization.

**CONCLUSIONS:**

By international standards, access to basic healthcare among older Brazilians is relatively high. Nevertheless, different levels of primary care problems between the public and private sectors and resulting utilization patterns suggest the need to continue working to close remaining gaps.

## INTRODUCTION

The Brazilian national health system, the *Sistema Único de Saúde* (SUS)^1^, is financed primarily through taxes with contributions from all levels of government. The SUS has reached nearly-universal levels of coverage and provides health services and common medications free of charge for all citizens, including those with a supplementary private health plans (currently approximately 26% of the population)^2^.

Since the early 1990s, the SUS has developed and scaled up community-based primary health care through the Family Health Strategy (FHS). The FHS provides primary care to a defined population via a multi-professional healthcare team with clinical protocols and national guidelines structuring actions on chronic disease and other health problems. As of 2016, the FHS included over 42,000 teams with more than 275,000 community health agents providing care to over 130 million persons, which represents approximately 62% of the Brazilian population^3^.

The FHS provides high levels of health care access^4^. The rapid expansion of the FHS is independently associated with improvements in several health outcomes, including a reduction in infant mortality^5–7^, a reduction in mortality from heart disease and in cerebrovascular deaths^8^, lower rates of ambulatory care sensitive hospitalizations^9^ ^,^ ^10^, and fewer complications from diabetes^11^. The program has decreased inequities in several types of healthcare access and utilization^12^ ^,^ ^13^ and contributed to enhance epidemiologic surveillance^14^.

Alongside the FHS, the SUS also offers primary care services via traditional health posts (which do not use multidisciplinary teams and usually do not include community health agents) to all Brazilian adults whose households are not registered with the FHS. A further 26% of the population obtains supplemental coverage through private health plans. These plans are highly varied and each health plan operator organizes services differently leading to different healthcare coverage, access, and quality by geographic region and type of health plan purchased^15^.

While several studies have discussed trends and disparities in healthcare access and utilization among older adults^16^, little is known about the primary care experiences of older Brazilians. This article responds to this need by describing national patterns of the primary care experiences of older individuals, assessing how these experiences differ by type of coverage, and then relating primary care experiences to overall patterns of ambulatory care utilization.

## METHODS

This study uses the baseline data from the ELSI-Brazil survey, a nationally representative, population-based cohort study of persons aged 50 years and older conducted between 2015 and 2016. The ELSI-Brazil sampling plan combined stratification of primary sampling units (municipalities), census tracts, and households. The final sample comprised 10,000 older adults (9,412 participated), residing in 70 municipalities from all five Brazilian geographic regions. Details of the ELSI-Brazil study^a^ have been described elsewhere^17^.

The main explanatory variable is the individual’s type of healthcare coverage. This is defined by those who have private health plans, those whose household was registered with the FHS, and those who have neither private health plan nor FHS coverage but are nevertheless covered by the traditional public health posts. The few respondents who answered they did not know if their household was registered with the FHS were categorized as having FHS coverage if they responded that they had received a community health worker visit anytime in the past two months.

Our measure of primary care experience was obtained from the respondent’s answers to a series of 12 questions regarding barriers to and quality of the person’s usual source of care (USC) (or most recent doctor visit among those with no USC). They include questions regarding access (is it easy to get an appointment, can an appointment be obtained in 24 hours, can appointments be made by phone), continuity of care (whether the person is seen by the same doctor, if the doctor knows what medications the patient is taking, if the doctor knows about the patient’s main health problems), communication (does the doctor listen, does the doctor explain things well, are visits long enough), and care coordination and resolution (does the doctor usually resolve the patient’s health problems, does the doctor speak about and help coordinate the patient’s specialist visits). All questions had the same Likert response format and were coded as to represent binary measures of poor primary care experiences (always or most of the time were categorized as zero and rarely or never were categorized as one). Based on the distribution among respondents, the total primary care problem score was then categorized into 0–2 problems, 3–4 (few) problems, and five or more (many) problems. We related these measures of primary care experiences to self-reported number of doctor visits broken down into general practitioner (GP) and specialist visits in the past 12 months. This analysis is intended to assess patterns of utilization among those with different types of healthcare coverage and to explore how primary care experiences affect the utilization of generalist and specialist services.

In the multivariable analyses, we controlled for factors that may contribute to the need for health services, including sex, age, the presence of any previously diagnosed chronic conditions (asthma, arthritis, cancer, depression, diabetes, heart disease, hypertension, and kidney disease), any functioning limitations (that is, any difficulty to carry out one or more activities of daily living), being in the lowest quintiles of a household wealth score (from a principal component analysis of a list of 16 common household goods), educational attainment, marital status (partnered versus others), and geographic region.

### Statistical Analysis

We present descriptive statistics as weighted proportions and, because we use complex survey data, statistical significance is obtained through an adjusted Wald test^18^. Estimates of prevalence rates are made using robust Poisson regression since some outcomes have prevalence rates of over 10%. Multivariable analyses of outcomes that involve counts (number of GP and specialist visits) are estimated via a hurdle regression model. This two-part model first assesses the probability of any event and, for those with at least one event, it then assesses the predictors of the intensity (number) of subsequent events^19^. All analyses control for the sample design of the survey and include final sample weights.

The ELSI-Brazil study was approved by the Research Ethics Committee of the Oswaldo Cruz Foundation, Minas Gerais (CAAE 34649814.3.0000.509).

## RESULTS

Table 1 presents the descriptive statistics by source of healthcare. Approximately 54% of the sample was female with an average age of 63 years. There were no demographic differences among individuals with different sources of healthcare and no differences in terms of chronic conditions. Nearly one quarter of the sample had a functional limitation with a significantly lower proportion among those with private health plans. As expected, those with private health plans were less likely to be in the lowest two (poorest) household wealth quintiles. Regarding health service utilization, 83% of the sample had at least one doctor visit in the past 12 months, ranging from 75% among those with traditional health units to 90% in those with private health plan coverage. The mean number of doctor visits among those who had at least one averaged approximately 4.4 and varied by group, in which the highest numbers were among those with private health plans. Among all doctor visits, GP visits were the highest among those registered with the FHS (79%) and the lowest among those with private health plans (47%), while specialist visits showed the opposite pattern. The intensity of GP and specialist visits was similar (slightly over three visits, on average), in which GP visits were higher in the public sector and specialist visits were higher among those with private health plans.

**Table 1:** Descriptive statistics and 95% confidence intervals, by source of care. Brazilian Longitudinal Study of Aging (ELSI-Brazil), 2015–2016.

Variable	Traditional health posts (n= 1,837)	Family Health Strategy (n = 5,356)	Private health plans (n = 2,210)	Total (n = 9,410)
Female	51.82 (47.62–56.01)	53.68 (50.53–56.79)	56.29 (51.80–60.68)	53.96 (51.00–56.90)
Age (mean)	62.78 (61.88–63.68)	62.49 (61.64–63.35)	64.26 (63.06–65.46)	62.99 (62.16–63.82)
Any functional limitation	26.16 (22.78–29.86)	27.65 (25.33–30.10)	20.73 (17.79–24.02)	25.62^a^ (23.57–27.78)
1 or more chronic conditions	72.69 (69.24–75.89)	74.22 (72.06–76.27)	73.95 (70.95–76.74)	73.86 (72.09–75.55)
Wealth quintiles (lowest 2)	38.51 (33.05–44.28)	43.54 (36.86–50.47)	15.82 (12.19–20.27)	35.75^a^ (30.63–41.21)
Doctor visit, past year	75.87 (72.61–78.86)	82.62 (80.64–84.43)	90.35 (88.40–92.01)	83.22^a^ (81.73–84.61)
Mean doctor visits^b^	3.85 (3.59– 4.12)	4.32 (4.10–4–54)	5.06 (4.78–5.34)	4.44^a^ (4.27–4.60)
1 or more GP visit, past year	66.88 (62.9–70.64)	79 (76.5–81.31)	46.85 (43.1–50.64)	68.27^a^ (65.79–70.65)
Mean GP visits^b^	2.96 (2.73–3.19)	3.51 (3.28–3.74)	3.31 (3.05–3.58)	3.38 (3.21–3.55)
1+ specialist visits, past year	63.52 (59.42–67.43)	55.73 (52.66–58.77)	87.84 (85.75–89.66)	65.71^a^ (63.4–67.94)
Mean specialist visits^b^	2.94 (2.61–3.26)	2.71 (2.51–2.90)	3.99 (3.71–4.27)	3.20^a^ (3.05–3.35)

GP: general practitioner or non-specialist doctor

^a^ p < 0.001 for difference among groups from design-corrected F test.

^b^ Among those with at least one visit.

Table 2 shows the distribution of primary care problems by main source of healthcare. Across all categories, those with private health plans tend to report the fewest primary care problems, followed by those registered with the FHS. Those who go to traditional health posts reported the most problems. All three groups reported similar levels of access problems with the exception of private health plan holders, who reported lower rates of problems in making appointments. In terms of problems with continuity of care, users of traditional health posts reported higher levels, while users of the FHS and private health plan holders reported similar results. The area of communication presented the lowest number of problems for all three groups, in which the largest difference was among those with private health plans, who reported lower levels of complaints regarding their appointment lasting enough time. Approximately half of the sample reported that their doctor does not discuss results of specialist visits. One area where the public sector outperformed the private is observed in the very low proportion of users of traditional health posts and FHS reporting no help from their provider in coordinating specialist visits. Finally, the overall number of primary care problems differed significantly among groups, in which private health plan holders averaged 2.7 problems, users of the FHS were at the mean with 3.5 problems, and users of traditional health posts were significantly above the mean with 4.5 reported problems.

**Table 2:** Primary care problems, by source of care^a^,b. Brazilian Longitudinal Study of Aging (ELSI-Brazil), 2015–2016.

Variable	Traditional public health posts (n = 1,837)	Family Health Strategy (n = 5,356)	Private health plans (n = 2,210)	Total (n = 9,410)
Access				
Difficult to make an appointment	0.5283 (0.4741–0.5818)	0.4031 (0.3736–0.4333)	0.2642 (0.2343–0.2965)	0.3926 (0.365–0.4209)
Cannot see doctor within 24 hours	0.6116 (0.5694–0.6522)	0.492 (0.4487–0.5354)	0.4486 (0.4058–0.4922)	0.5043 (0.4715–0.537)
Difficult to get information by phone	0.732 (0.6881–0.7718)	0.6359 (0.5967–0.6735)	0.4361 (0.4076–0.4652)	0.6043 (0.5742–0.6336)
Continuity of care				
Rarely/never see the same doctor	0.4416 (0.4123–0.4712)	0.3738 (0.3435–0.4051)	0.2612 (0.2338–0.2906)	0.3588 (0.3362–0.3821)
Doctor doesn’t know the patient’s medications	0.2589 (0.2292–0.291)	0.1801 (0.1609–0.2011)	0.1116 (0.0925–0.1341)	0.1777 (0.1633–0.1932)
Doctor doesn’t know the patient’s health problems	0.3002 (0.2718–0.3301)	0.1798 (0.1559–0.2064)	0.1208 (0.1002–0.145)	0.188 (0.1677–0.2102)
Provider communication				
Doctor does not listen well	0.2542 (0.2217–0.2896)	0.173 7(0.1551–0.1941)	0.1099 (0.0909–0.1322)	0.1732 (0.158–0.1896)
Doctor does not explain things well	0.2732 (0.2439–0.3046)	0.2056 (0.1842–0.2287)	0.1296 (0.1061–0.1574)	0.1996 (0.1838–0.2165)
Appointment does not last enough time	0.3333 (0.3076–0.3601)	0.2763 (0.2473–0.3073)	0.1796 (0.1569–0.2049)	0.2632 (0.2433–0.2841)
Coordination and care resolution				
Doctor does not discuss a specialist visit	0.573 (0.5356–0.6096)	0.473 (0.4519–0.4942)	0.4566 (0.4296–0.4838)	0.4881 (0.4691–0.5073)
Little/no help making a specialist appointment	0.0517 (0.0377–0.0704)	0.0326 (0.0237–0.0447)	0.1742 (0.1402–0.2143)	0.0718 (0.0598–0.086)
Doctor unable to resolve the patient’s health problems	0.337 (0.3049–0.3706)	0.2545 (0.2344–0.2758)	0.1456 (0.1233–0.1712)	0.2433 (0.2262–0.2613)
Mean primary care problems (of 12)	4.46 (4.18–4.73)	3.52 (3.30–3.74)	2.73 (2.54–2.92)	3.51 (3.33–3.69)

^a^ Figures are survey-weighted proportions and their respective 95% confidence intervals.

^b^ For all variables, differences across groups are statistically significant (p < 0.001).

**Figure 1:**
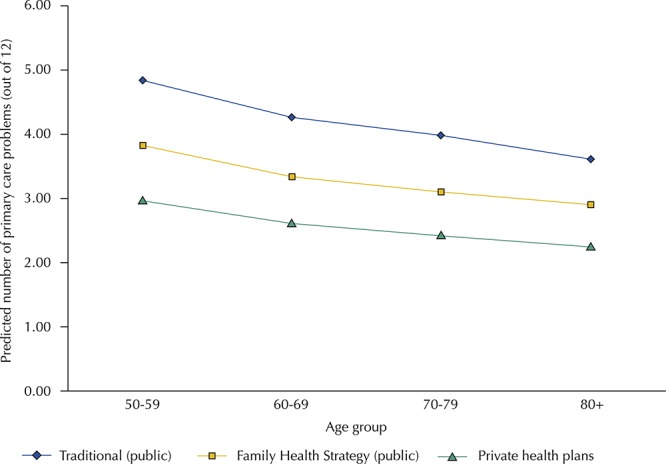
Relation between number of primary care problems and source of care by age group*. Brazilian Longitudinal Study of Aging (ELSI-Brazil), 2015–2016.

Figure 1 illustrates the relation between the total number of primary care problems, type of healthcare coverage, and age. It shows that, once important determinants of healthcare need such as demographics and socioeconomic status are controlled for, the total number of primary care problems declines with age, but it was consistently the highest for those in traditional health units. Those registered with the FHS had lower levels than users of traditional health units, but they had higher numbers of problems at each age group than those with private health plans.

* Predicted number of primary care problems from survey-adjusted Poisson regression that additionally controls for sex, age, household wealth, educational attainment, civil status, chronic conditions, functional limitations, and region of the country. Differences between traditional, FHS, and private plans are statistically significant (p < 0.001).

Table 3 presents the results from the multivariable hurdle regression models. For GP visits, women were more likely than men to have any visit but not to report a higher number of doctor visits in the past 12 months. Those registered with the FHS were 62% more likely and those with private health plans were 48% less likely to have a GP visit than those using traditional health posts. Among those with at least one GP visit, being a user of the FHS was associated with higher intensity of GP utilization. A greater number of primary care problems was positively associated with GP visits but negatively associated with their intensity, while health problems (both functional limitations and chronic conditions) had a large positive association with both outcomes. Finally, those in the lowest wealth quintiles had a higher likelihood of any GP visit but not for the total number of GP visits.

**Table 3:** Factors associated with healthcare utilization^a^. Brazilian Longitudinal Study of Aging (ELSI-Brazil), 2015–2016. (n = 6,445)

Variable	Any GP visit	Number of GP visits	Any specialist visit	Number of specialist visits
Family Health Strategy (versus traditional health post)	1.62^c^ (1.35–1.96)	1.13^b^ (1.03–1.25)	0.81^b^ (0.67–0.98)	1.01 (0.88–1.15)
Private health plan	0.52^c^ (0.41–0.66)	1.1 (0.93–1.29)	3.06^c^ (2.32–4.04)	1.51^c^ (1.30–1.75)
3–4 primary care problems (versus < 3)	1.09 (0.92–1.28)	0.98 (0.87–1.10)	0.85 (0.71–1.03)	0.94 (0.81–1.08)
≥ 5 primary care problems	1.36^c^ (1.18–1.56)	0.8^c^ (0.71–0.90)	0.73^c^ (0.63–0.84)	0.9^c^ (0.79–1.02)
Female	1.22^b^ (1.02–1.47)	1.13 (1.00–1.27)	0.98 (0.83–1.16)	1.09 (0.97–1.22)
Any functional limitation	1.02 (0.83–1.26)	1.37^c^ (1.20–1.56)	1.52^c^ (1.32–1.76)	1.33^c^ (1.18–1.50)
1 or more chronic conditions	1.21^b^ (1.03–1.43)	1.59^c^ (1.41–1.80)	1.24^c^ (1.04–1.48)	1.37^c^ (1.21–1.55)
Lowest 2 household wealth quintiles	1.27^b^ (1.06–1.53)	1.02 (0.90–1.15)	0.63^c^ (0.53–0.74)	0.93 (0.79–1.10)

GP: general practitioner or non-specialist physician

^a^ Numbers are prevalence ratios and 95% confidence intervals from hurdle regression analyses that additionally control for age, educational attainment, civil status, and region of the country.

^b^ p < 0.05

^c^ p < 0.001

Regarding specialists, those registered with the FHS were less likely than users of traditional health posts to have any specialist visit, while those with private health plans were more likely than the other two groups to have any specialist visit and to visit specialists more frequently. Those with greater primary care problems had a lower likelihood of both categories of specialist visits, while having a functional limitation or chronic condition raised this likelihood. Those in the lowest wealth quintiles were less likely to have had any specialist visit.

**Figure 2:**
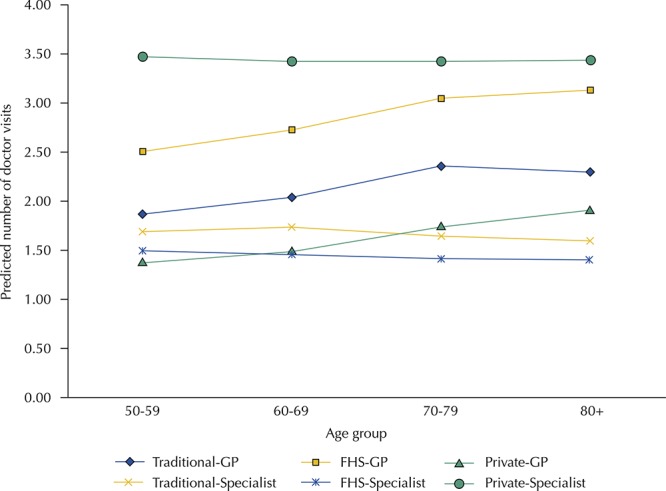
Predicted number of GP (general practitioner) and specialist doctor visits by age group and source of healthcare*. Brazilian Longitudinal Study of Aging (ELSI-Brazil), 2015–2016.

Figure 2 presents the predicted numbers of GP and specialist visits by age group and source of care, controlling for determinants of healthcare need. For GP, utilization increased with age for all types of healthcare. By age 80, rates of GP use among users of the FHS and private health plan holders were equal to four visits/year and those using traditional health posts had lower rates (3.2). Specialist visits did not increase with age, but private health plan holders had approximately two-thirds greater use of specialists than those who used only the public sector.

FHS: Family Health Strategy

* Data are predicted numbers of doctor visits among those with at least one visit in the past 12 months from Poisson regression controlling for demographic, socioeconomic, and health-related variables as presented in Table 3.

## DISCUSSION

The study has shown that, among a nationally-representative cohort of older Brazilians, levels of access to and use of basic health services were quite high: approximately 83% had at least one doctor visit in the past 12 months, with an average of 4.4 visits per year. Those with private health plans most frequently visited specialists, while those in the Family Health Strategy and traditional health posts were more likely to visit a general practitioner. Primary care experiences differed significantly with the highest number of problems experienced among those using traditional health posts. These primary care experiences were in turn associated with ambulatory care utilization and its intensity.

These results suggest that healthcare experiences among older Brazilians are strongly influenced by the type of healthcare coverage. Some of these differences are likely due to selection into private health plans. Previous literature documents important demographic, epidemiologic, and socioeconomic differences between those with private health plans and those who exclusively use the SUS^4^ ^,^ ^15^ ^,^ ^20^. This study confirmed that older individuals with private health plans were significantly less likely to be in the lowest household wealth quintiles and had lower rates of functional limitations, but they did not differ from the rest of the population in relation to chronic conditions. Differences in primary care experiences and patterns of healthcare utilization among those with private health plans persisted even after controlling for healthcare needs and socioeconomic status, which suggests that there are likely important unmeasured factors that differentiate those with private health plans from those without them.

Perhaps more importantly, there were significant differences in primary care experiences between older individuals covered by the two different types of primary care offered through the SUS. While these differences have been documented previously^4^ ^,^ ^20^, the implications for older adults are significant as worse experiences in terms of primary care access, continuity, communication, and care coordination can affect chronic disease management and worsen the quality of life.

To provide an international perspective on the results of this study, we compared results observed here with those in several other contexts. For example, in the US, where older Americans are largely covered by government insurance, 90% of older Americans report having made a doctor visit in the past two years^21^. In Mexico, where older persons are covered by several different mechanisms, doctor visits among those with a health problem in the past two weeks ranged from 55% among those uninsured to 78% among those with social security coverage^22^. In a European cohort study of aging (SHARE), among older adults in 15 European countries, only 15% had no health service utilization in the past 12 months, 86% had a GP visit, and 40% had a specialist visit^23^. Thus, the observed rates of healthcare use in Brazil – and the differences observed among those with different sources of healthcare – are largely in line with those observed among older populations in other middle and higher-income country contexts.

There are several implications of the study results. First, like most health systems around the world, the SUS struggles to meet the changing needs of its older population. Complications resulting from chronic underfunding have led to slow adoption of technology at the primary care level^24^ and uneven access and coverage of the SUS in some areas. These deficiencies could be addressed through adaptation of existing programs such as the National Program for Improving Primary Care Quality and Access (PMAQ)^25^. Existing PMAQ protocols and targets could be adapted to explicitly address and stimulate improved primary care quality for older adults.

Second, the decentralized nature of Brazilian healthcare management provides a number of advantages but has also led to a complex and varied health service settings with only some municipalities reaching universal FHS coverage. Given the important differences in access, continuity of care, provider communication, and care coordination observed between the FHS and traditional health posts, the latter’s continued transformation into the FHS model should be a national priority.

Third, although private health plan holders had better financial and physical health than those using the SUS, their high use of specialist services indicates that private health plan operators need to consider how a stronger primary care orientation may provide a more balanced and coordinated approach to healthcare for older adults. Furthermore, the high number of private sector specialist visits is not only likely to reflect higher costs in that sector (which implies higher government expenditures on health given the existing generous public tax subsidies for those who purchase private health plans), but it also has implications for human resources in the SUS, given healthcare shortages in specialty and diagnostic services experienced throughout the public sector^26^.

Finally, there is a continued need to update and adapt the model of healthcare delivery (in both the public and private sectors) to better meet population needs. To date, most of the care provided has been focused on the identification and management of only a few chronic conditions and this approach does not yet reflect a whole person orientation required for the promotion of healthy aging. For these reasons, the new *caderneta de saúde para a pessoa idosa* (health notebook for the older person) along with other interventions are urgently needed to accelerate the process of adapting the Brazilian health system to meet the healthcare needs of the country’s rapidly aging population.

## Footnotes

a Fundação Oswaldo Cruz. Brazilian Longitudinal Study of Aging. Rio de Janeiro; c2015 [cited 2017 Nov 28]. Available from: http:// elsi.cpqrr.fiocruz.br
